# Impact of Intensive Land-Based Fish Culture in Qingdao, China, on the Bacterial Communities in Surrounding Marine Waters and Sediments

**DOI:** 10.1155/2011/487543

**Published:** 2011-09-13

**Authors:** Qiufen Li, Yan Zhang, David Juck, Nathalie Fortin, Charles W. Greer

**Affiliations:** ^1^Yellow Sea Fishery Research Institute, Chinese Academy of Fishery Science, 106 Nanjing Road, Shandong, Qingdao 266071, China; ^2^Biotechnology Research Institute, National Research Council of Canada, 6100 Royalmount Avenue, Montreal, Québec, Canada H4P 2R2

## Abstract

The impact of intensive land-based fish culture in Qingdao, China, on the bacterial communities in surrounding marine environment was analyzed. Culture-based studies showed that the highest counts of heterotrophic, ammonium-oxidizing, nitrifying, and nitrate-reducing bacteria were found in fish ponds and the effluent channel, with lower counts in the adjacent marine area and the lowest counts in the samples taken from 500 m off the effluent channel. Denaturing gradient gel electrophoresis (DGGE) analysis was used to assess total bacterial diversity. Fewer bands were observed from the samples taken from near the effluent channel compared with more distant sediment samples, suggesting that excess nutrients from the aquaculture facility may be reducing the diversity of bacterial communities in nearby sediments. Phylogenetic analysis of the sequenced DGGE bands indicated that the bacteria community of fish-culture-associated environments was mainly composed of Flavobacteriaceae, gamma- and deltaproteobacteria, including genera *Gelidibacter, Psychroserpen, Lacinutrix,* and *Croceimarina*.

## 1. Introduction

Land-based intensive fish culture is developing at a high speed in China and has brought about the fourth mariculture fervor in recent years. While it is worth paying attention to the discharge of large quantity of untreated effluent, La Rosa and coworkers [1] have reported that in oligotrophic marine environments, addition of various nutrients through feed, detritus, and fecal matter can induce changes in the macro-, meio-, and micro-fauna community structure in the water column and sediment. Moreover, it has been proved that intensive fish husbandry often lead to environmental eutrophication, foreign species, and disease introduction [2, 3]. In 2006, intensive fish farming in the Philippines was demonstrated to be detrimental to the reef-building coral *Pocillopora damicornis*, since many biological aspects of coral were impaired by exposure to effluent from fish farms [4]. 

Bacterial communities play important roles in nutrient circulation and are sensitive to changes of environment. For example, accumulation of large amounts of organic matters can induce persistent alterations in bacterial assemblage [5]. Comprehensive characterization of microbial populations in regions adjacent to aquaculture operations is important for the prevention and treatment of various diseases of farmed fish and for the maintenance of water quality [6]. However, traditional culture-dependent approaches are time-consuming and costly, and the data cannot represent actual situations, as ~99.99% of the microorganisms in the natural environment are currently uncultivable [7]. Therefore, the composition of bacteria in aquaculture ecosystems is very poorly understood [8]. To our best knowledge, by far, there are few reports on the composition and structure of bacteria community associated with land-based intensive fish culture and the impact of such fish-culture performance to the nearby environment. 

In recent years, many molecular biological approaches have been successfully applied to microbial ecology analysis, for example, denaturing gradient gel electrophoresis (DGGE), which was originally developed for analyzing gene mutation based on the sequence difference of PCR products by electrophoresis in the medicine research field. It was first applied by Muyzer et al. [9] to study the diversity of microbes in 1993. Since then, DGGE has been widely used in microbial diversity analyses of different ecoenvironments, such as explosive-polluted soil [10], estuary [11], scallop early stage environment [12], shrimp guts [13], and the offshore cage fish farms [6]. However, it has never been utilized to examine the microbes in land-based fish-culture-associated environment. In this study, bacterial community composition of the environment associated with intensive land-based marine fish culture was investigated through culture-dependent and culture-independent approaches, with the aim to characterize the bacteria compositions of associated environment and to evaluate the effect of intensive fish culture on the bacteria community in nearby sea areas.

## 2. Materials and Methods

### 2.1. Description of Sites and Sampling

The intensive land-based fish-culturing farm is located in the suburb of Qingdao, China. It is a newly developed industry and mainly raises turbot (*Scophthalmus maximus*) and Japanese flounder (*Paralichthys olivaceus*) with natural and underground sea water. Most of the untreated effluent is discharged into the nearby Aoshan Bay. The samples were collected from the fish farming ponds, effluent channel, polluted sea areas 10 m off the effluent channel end, and unpolluted sea area 500 m off the channel end. Triplicate samples were collected with sterilized containers from each site, each including 2 L of sea water and 50 g of sediment, and were transferred to the laboratory on ice in time. Subsamples were then treated for bacteria cultivation. The remaining samples were stored at −20°C for molecular analysis.

### 2.2. Detection of Bacteria Groups with Different Physiological Functions

The sediment and water samples were serially diluted 10-fold with sterilized sea water, and 0.1 mL aliquots of the dilution were spread onto Zobell's 2216E medium for heterotrophic bacteria and other appropriate media for ammonia oxidizing bacteria and nitrifying bacteria [8]. Colonies on the plates were counted after 2-3 days incubation at 28°C. 

Sulfate and nitrate reducing bacteria were detected with “Most Probable Number” method as described previously [6, 14]. Briefly, aliquots of 1 mL series dilution were added into series 10 mL of media. Triplicate tubes were prepared for each dilution. Cultures were detected after 7 days of incubation at 28°C for nitrate reducing bacteria and 14 days for sulfate reducing bacteria. The population size of bacteria was calculated by referring to the table, according to the tubes with positive outcomes at each dilution. The three counting results for each sampling site were averaged, and the standard deviations (STDEV) were shown.

### 2.3. Extraction of Genomic DNA of Bacteria

Genomic DNA was extracted using the chemical-enzymatic lyses protocol [15] with a few modifications. Briefly, the membrane for water sample or 10 g of each sediment sample with 5 mL of sterilized distilled water were vortexed at maximum speed for 5 min, then 1 mL lysozyme (100 mg/mL) and 4 mL DNA extraction buffer (100 mM Tris-HCl, 100 mM EDTA, 100 mM Na_3_PO_4_, 1.5 M NaCl, pH 8.0) were added to the tubes. The samples were incubated on shaking inoculators for 1 h at 30°C, and another 1 h at 37°C after 20 *μ*L proteinase K (100 mg/mL) was added, followed by 5~15 min at 85°C with 100 *μ*L 20% SDS. Subsequently, samples were then centrifuged at 4,100 g for 15 min. One-second volume of 7.5 M ammonium acetate was added to the supernatants, followed by incubation on ice for 15 min. Thereafter, tubes were centrifuged at 4°C and 9, 400 g for 15 min, and the supernatants were treated with cold 2-propanol overnight at −20°C. Pellets were raised with 70% and 95% ethanol, respectively. DNA was finally resuspended in sterilized distilled water. The crude DNA extract was purified with polyvinylpolypyrrolidone (PVPP) and sephacryl S-400 spin columns as described by Elliott [2] to remove PCR inhibitors, such as humid acid. Untreated and treated DNA were compared by electrophoresis 0.7% agarose gel at 60 V for 2 h and visualized on a MultiImage light Cabinet (Alpha Innotech Corporation, France).

### 2.4. Amplification of 16S rDNA

The bacterial universal primers, U341 and U758, were used to amplify a 418 bp fragment corresponding to position 341 to 758 bp of *Escherichia coli* 16S rDNA sequence [9]. To stabilize the melting behavior of the amplified fragments in the DGGE reaction, the forward primer contained a GC-clamp [10]. Sequences of the U341 and U758 were as follows: U341: 5-GCGGGCGGGGCGGGGGGCACGGGGGGCGCCGGC-GGGCGGGGCGGGGGCCTACGGGAGGCAGCAG-3′; U758: 5′-CTACCAGG GTATCTAATCC-3′. For optimum DGGE result, different PCR conditions were tested. The optimum PCR reaction was carried out in a 50 *μ*L volume, including 5 *μ*L of genomic DNA as the template, 5 *μ*L 10 × PCR buffer, 25 pmol of each primer, 200 *μ*M of each dNTP, 1 mM MgCl_2_, and 2.5 units of Taq polymerase (Amersham Biosciences, Piscataway, USA). Before adding Taq polymerase, samples were denatured at 96°C for 5 min, followed by a touchdown PCR protocol [15] in which the annealing temperature was set to 65°C and decreased by 1°C every cycle until it reached 55°C. Each cycle included denaturation at 94°C for 1 min, anneal for 1 min, and extension at 72°C for 3 min. Twenty additional cycles were carried out with annealing at 55°C. Finally, 5 *μ*L of each PCR product was loaded onto a 1.4% agarose gel with a 100 bp DNA ladder (MBI Fermentas, Amherst, USA). Bands were visualized with SYBR safe dye in the MultiImage light cabinet.

### 2.5. DGGE Analysis of Amplified DNA

DGGE was performed on the Decode Universal Mutation Detection System (Bio-Rad Inc., Mississauga, Canada) as described by the manufacturer. The separation was carried out on an 8% (W/V) acrylamide gel in 1X TAE (40 mM Tris-acetate, pH 8.0; 1 mM Na_2_DETA) containing a linear gradient from 25% to 65% denaturant (100% denaturant consisted of 7 M urea and 40% formamide) as described by Muyzer et al. [9]. To avoid disturbance of the gradient during comb insertion, a 6% acrylamide-N,N-methylene: bisacrylamide (37.5 : 1) stacking gel without denaturant was added [15]. Each purified PCR product (about 600 ng) with 15 *μ*L of 2X loading buffer was applied to one lane of the denaturing gradient gel. The electrophoresis was run for 16 h at 80 V, then stained in 1 : 10000 dilution of Vistra Green staining solution (Amersham Pharmacia Biosciences Inc., Baie-d'Urfe, Canada) for 30 min, and visualized on a FluorImager system (Model 595, Amersham) with a 488 nm excitation filter and a 530 nm emission filter. 

To analyze the bacterial diversity, the Shannon index of each sample was calculated according to the strength (shown as the absorbance) and position of the DGGE bands in every lane, and (1) was used

(1) $$\begin{array}{c} H=-\sum \left(\frac{n_{i}}{N}\right)\text{lg}\left(\frac{n_{i}}{N}\right). \end{array}$$

In (1), *n*_*i*_ means the area of absorbance peak of each band and *N* means the total area of absorbance peak of all bands in a lane. 

 Dendrogram analysis of DGGE band patterns was performed using the Dendron 2.2 software package (Soll-tech Inc., Oakdale, USA). The unweighted pair group method, based on a similarity matrix calculated from the presence/absence of DGGE bands, was used to analyze the similarity between the samples.

### 2.6. Reamplification and Sequencing of DGGE Bands

From the gels, 32 specific DGGE bands were excised with a sterile surgical scalpel. DNA from these bands was eluted by incubating overnight at 37°C in sterilized deionized water [16] and then purified with QIA quick PCR purification kit (Qiagen, Mississauga, Canada). The obtained DNA was used as template for reamplification. The standard PCR was performed in a 50 *μ*L reaction volume, containing 1 *μ*L DNA, 1 *μ*L U341 primer (25 pmoL), 1 *μ*L U758 primer (25 pmoL), 0.625 *μ*L BSA (10 mg/mL), 5 *μ*L 10X PCR buffer, 8.0 *μ*L MgCl_2 _(100 mg), 8.0 *μ*L dNTPs (1.25 mM), 24.9 *μ*L sterile deionized water, and 0.5 *μ*L Taq polymerase which was added separately when the temperature reached 80°C after initial denaturization for 5 min at 95°C. The PCR included 25 cycles of 1 min at 94°C, 1 min at 64°C, and 1 min at 72°C. In order to get single bands for clean sequencing results, the quantity of template, annealing temperature, and cycle number were adjusted according to the result of standard PCR protocol for individual samples. Amplicants showing single bands in a 1.4% agarose gel were purified with GFX Purification Kit (Amersham, Piscataway, USA) and quantified by loading 1 *μ*L onto a 1.4% agarose gel in comparison with dilution series of 100 bp DNA ladder. Samples (20 *μ*L, 2 ng/*μ*L) were sent to Laval University for sequencing.

### 2.7. Phylogenetic Analysis of Bacterial Communities

The obtained sequences were manually corrected by comparing the consensus of forward and reverse sequences with software Macvector 8.1 (MacVector Inc., Cary, USA). The length of the corrected sequences varied in the range from 352 to 387 bp. The sequences were initially aligned using the Clustal W program, then they were analyzed referring to the closely related sequences retrieved from the NCBI website: http://blast.ncbi.nlm.nih.gov/Blast.cgi?PROGRAM=blastn. Identical sequences with the same migration on DGGE were treated as one. Further manual amendments to the alignment were performed using the multicluster function.

## 3. Results

### 3.1. Number of Bacteria Detected with the Culture-Dependent Method

Bacteria from 5 important physiologically defined groups were found in all sediment and water samples. As shown in Table 1, the counts for total heterotrophic bacteria in the fish pond and effluent channel were the highest (1.25 to 1.29 × 10^5^ CFU/g), followed by polluted sea areas accepting fish culture effluent (1.23 to 4.7 × 10^4^ CFU/g), and that of unpolluted sea areas 500 m off the effluent channel was the lowest (1.6 to 4.3 × 10^3^ CFU/g). Bacteria numbers in sediment were all higher than those of related water environments. The numbers of ammonium-oxidizing bacteria, nitrifying bacteria, and nitrate-reducing bacteria showed similar distribution trend to heterotrophic bacteria, varied from 4.0 × 10^1^ cells/g to 1.5 × 10^5^ cells/g, suggesting active nitrogen circulations in the polluted areas. The numbers of sulfate-reducing bacteria, however, were only 2.3 × 10^1^ cells/g to 4.6 × 10^2^ cells/g in the sediments and 3*∼*7.5 × 10^1^ cells/g in the waters, significantly lower than those of other bacteria. 

### 3.2. Genomic DNA of Bacteria Isolated from Fish-Culture-Associated Environments

**Figure 1:**
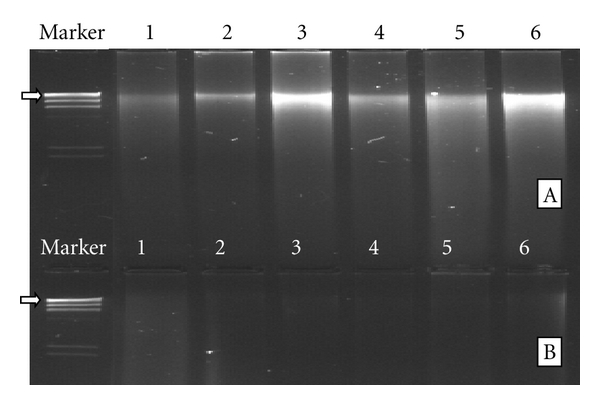
Electrophoresis of genomic DNA isolated from bacterial community in water and sediments of fish-culture-associated environments in Qingdao, China. Showing PVPP and Sephacryl is effective to purify the crude DNA extracts. (A) purified DNA extracts with PVPP and Sephacryl; (B) crude DNA before purification. Marker: *λ*DNA digested with HindIII (arrow indicates a 23.1 kb fragment), 1: water from the fish culture pond; 2: water in effluent channel; 3: water from polluted sea area; 4: sediment from polluted sea area; 5: water from unpolluted sea area; 6: sediment from unpolluted sea area.

The size of obtained Genomic bacterial DNA fragment was about 23 kb. The extracts became colorless from brown, and their electrophoresis bands became much clearer after purification with PVPP and Sephacryl (S-400) columns (Figures 1(A) and 1(B)), indicating that PVPP and Sephacryl purification were effective in removing inhibiting factors in the crude extracts.

### 3.3. Amplification of 16S rDNA

**Figure 2:**
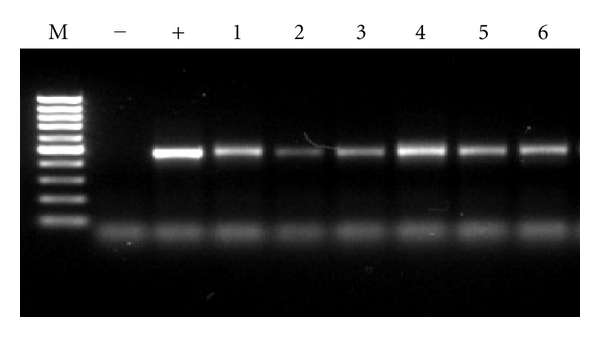
Gel electrophoresis of PCR-amplified 16S rDNA of genomic DNA, indicating the target DNA fragment was successfully amplified in the 6 samples. M: 100 bp DNA ladder; −: negative control; +: positive control; 1; water from the fish-culture pond; 2: water in effluent channel; 3: water from polluted sea area; 4: sediment from polluted sea area; 5: water from unpolluted sea area; 6: sediment from unpolluted sea area.

A 417 bp fragment of 16S rRNA gene was amplified with primers GC U341 and U758. The touch-down protocol insured single-specific bands. The yield was reasonably high, as the bright bands shown in Figure 2. 

### 3.4. DGGE Band Profiles of Samples from Various Environments

**Figure 3:**
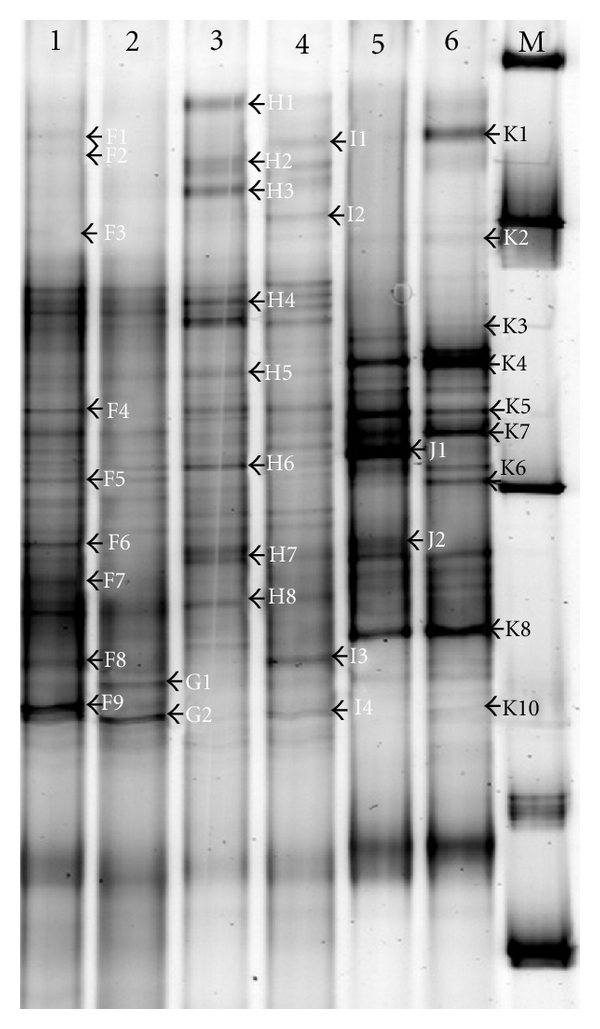
DGGE profiles of 16S rDNA fragments of bacterial communities in various water and sediment samples, showed the high diversity in fish culture associated environment and the difference between samples. 1: water from polluted sea area; 2: water from unpolluted sea area; 3: sediment from polluted sea area; 4: sediment from unpolluted sea area; 5: water from the fish culture pond; 6: water from effluent channel; M: Marker.

DGGE analysis of PCR products produced identical patterns, with more than 20 bands for each sample, indicating a high diversity of bacteria community. As shown in Figure 3, band patterns of sediments showed higher diversity and more homogenized distribution than that of waters. The bacterial diversity in water reduced with the increase of the distance from fish ponds. This can be demonstrated by their Shannon index, as shown in Table 2. The significance of dominant bands in these samples also differed greatly, with water from fish ponds > water from effluent channel > water from polluted sea area > water from unpolluted sea area > sediment of polluted sea area > sediment of unpolluted sea area, suggesting that fish culture could lead to a reduction of bacterial diversity and some species could become absolutely dominant. 

**Figure 4:**
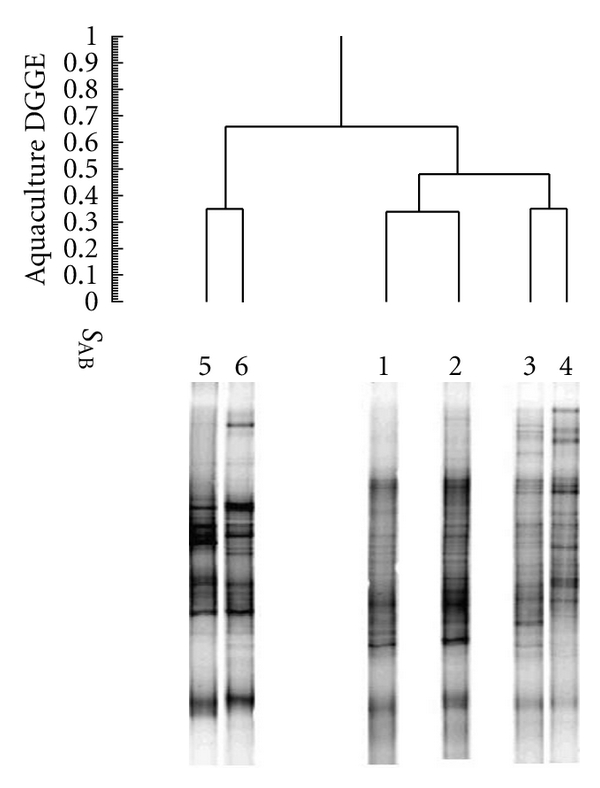
UPGMA dendron similarity assessment of the DGGE profile illustrated in Figure 3 showed the similarity between samples. 1: water from polluted sea area; 2: water from unpolluted sea area; 3: sediment from polluted sea area; 4: sediment from unpolluted sea area; 5: water from the fish-culture pond; 6: water from effluent channel.

Dendrogram of the DGGE band patterns reflected the correlation/similarity of different DGGE lanes. As shown in Figure 4, the two sediment samples from polluted and unpolluted sea area were clustered into one group (S_AB_, 0.67) and were clustered into one big group with the two water samples from the same sea area (S_AB_, 0.52). However, the two water samples from fish ponds and effluent channel were clustered into the other group (S_AB_, 0.65). Meanwhile, the similarity coefficient (S_AB_) of samples from fish culture pond and samples from sea area was only 0.34, suggesting that composition of bacterial communities in the same habitat was more similar. 

### 3.5. Phylogenetic Analysis of Sequenced DGGE Bands

In total, 32 bands in DGGE gel were selected and reamplified with the primers U341 and U758. Among them, 19 produced clean sequencing results. The closest matches of these sequences were then identified by NCBI BLAST analysis. Results were summarized in Table 3. The similarity of these sequences compared to references in database ranged from 93% to 100%. 

**Figure 5:**
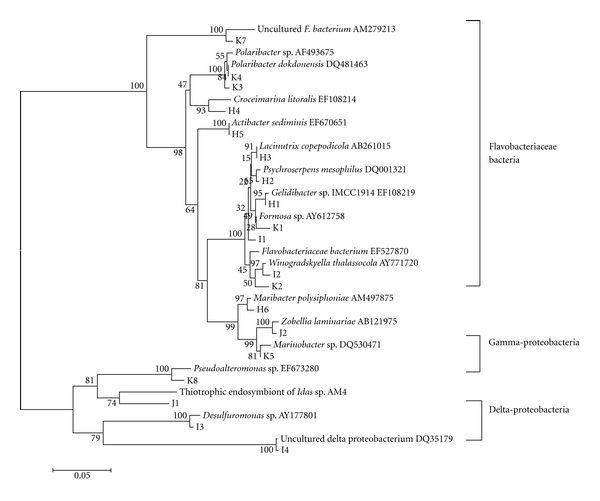
Phylogenetic tree of the sequences of 16S rDNA fragments separated by DGGE showed the classification positions of the bacteria and the phylogenetic relationship between each other. Reference sequences are shown with their respective Genbank accession numbers. The tree was built by MEGA bootstrap 1000 using neighbor joining.

Phylogenetic analysis of the sequences revealed the bacterial community structure of the land-based fish-culture-associated environments. In general, the communities were composed of Flavobacteria, Gammaproteobacteria and Deltaproteobacteria. Among them, Flavobacteria showed strong dominance, and it covered genera *Gelidibacter, Psychroserpen, Lacinutrix, Croceimarina, Actibacter, Maribacter, Winogradskyella, Zobellia, Formosa, *and* Polaribacter. *The two proteobacteria groups ranted a small part of the total population. Some of these species had not been cultured independently, such as I4 and K7. For individual environment, *Polaribacter *sp. (K3, K4),* Marinobacter *sp. (K5), thiotrophic endosymbiont of *Idas *sp. (J1), and* Pseudoalteromonas* sp. like bacteria (K8) were dominant in fish culture effluent and polluted sea water samples. In addition, *Formosa* sp. (K1) was also found to be significantly dominant in the polluted sea water. On the other hand, dominant bacteria in sediment samples of polluted sea area were not as significant as that in water samples. These dominant bacteria in sediment were composed of *Gelidibacter *sp. (H1),* Lacinutrix copepodicola *(H3),* Croceimarina litoralis *(H4), and *Maribacter polysiphoniae *(H6). The unpolluted sea area contained almost all bacteria species in the above-mentioned environments and distributed evener than them. At the same time, some unique species, such as *Winogradskyella thalassocola *(I2),* Desulfuromonas* sp. (I3), and uncultured delta proteobacterium (I4), presented in the unpolluted sea area. The phylogenetic relationship of the above-mentioned bacteria is shown in Figure 5.

## 4. Discussion

The feed conservation ratio of intensively cultured fish was reported to be 71.2–74.9%, and the faeces production ratio was 9.6–3.1%. These implied that 133 kg of nitrogen and 28.8 kg of phosphorous would be discharged into the environment for 1 ton of fish [17]. Discharge of detritus and fecal matters produced due to the addition of feed, to the oligotrophic marine environment can induce changes in the community structures of macro-, meio- and microfauna in the water columns and sediments [1], as well as the nutrient level, physical, and chemical conditions. The five physiologically defined bacteria groups chosen in this study have close relationship with the content of organic matter, levels of dissolved oxygen, and nitrogen and sulfur circulation activities in their environment. The results of our study showed that the counts for heterotrophic bacteria gradually reduced with the increase of distance from the fish ponds, suggesting that fish culture effluent could introduce abundant organic matters and heterotrophic bacteria to the sea area accepting it. The high numbers of ammonium-oxidizing bacteria, nitrifying bacteria, and nitrate-reducing bacteria in the effluent water and polluted sea area indicated active nitrogen circulation in these areas. This could be attributed to the abundant nitrogen brought forth by fish-culture effluent with fish metabolic excreta (feces, etc.) and waste feeds. 

Yoza et al. [6] observed similar DGGE gradient profiles for a newly developed cage fish-culture sediment sample and a 300 m upcurrent control sample. However, they still expected that sufficient nutriment addition would impact the sediment environment. In our experiments, less diversity and evenness in species distribution was observed from sediment samples in polluted sea areas than that in unpolluted sea areas, with Shannon index 1.37 and 1.46, respectively. These observations proposed that intensive land-based fish-culture effluent have produced significant impact on the bacteria community, leading to reduction in bacterial diversity. Furthermore, both of the studies were carried out shortly after the development of fish culture. With a longer culturing time, it is reasonable to believe that the impact would be much more significant. Asami et al. [18] also reported that intensive shellfish aquaculture accelerated sulfur cycle in the beneath coastal marine sediment [17]. Moreover, the bacterial community was decided by the habitat rather than by its geographic location [19]. Namely, the impact of fish-culture effluent to the bacterial communities may occur by changing the chemical and physical conditions of their habitat, besides importing bacteria from effluent. 

One of the dominant phylotype (K8) found in the fish culture effluent and polluted sea water area belonged to the genus, *Pseudoalteromonas* of Gammaproteobacteria. This genus had a widespread distribution in the marine environment [20]. It was reported that *Pseudoalteromonas* had both deleterious and beneficial effects on marine eukaryotes [21–23]. The dominant phylotype (I3) in the sediment of unpolluted sea area was similar to *Desulfuromonas sp.*, a sulfate-reducing bacterium in *Delta-*proteobacteria family. It was not surprising to find sulfate reducers in the marine sediment, since sulfate is a favored terminal electron acceptor in this environment [24], though the number of bacteria detected was very low through culture-dependent methods in this paper. Genus *Formosa* (K1) was found both in the effluent channel water and native sea water, and it was a heterotrophic, gram-negative, motile, aerobic, and brown alga-degrading bacterial group [25, 26], indicating its commitment to the marine environment. 

Many of the main bacteria groups, such as *Aeromonadaceae, Pseudomonadaceae,* and *Vibrionaceae* detected as pathogens of farmed fish with traditional culture-depended methods, were not detected by molecular methods in this paper, suggesting that pathogenic bacteria might not be dominant in the whole community. So, the bacterial composition is still far more complex than we could imagine. Further study is necessary to determine whether and how long the aquaculture could change the composition and destroy balance of bacterial communities in its nearby sea area.

## 5. Conclusion

In the present paper, the impact of intensive land-based fish culture in Qingdao, China, on the bacterial communities in surrounding marine environment was analyzed through culture-based and molecular-based approaches. The result of culture-based studies showed that counts of heterotrophic, ammonium-oxidizing, nitrifying, and nitrate-reducing bacteria reduced with the distance increasing from fish ponds to the unpolluted sea area. DGGE profiles showed fewer bands in the samples taken from near the effluent channel compared with more distant sediment samples. All the above suggested that excess nutrients from the intensive land-based fish culture facilities may import bacteria to and change the chemical and physical conditions of the nearby sea area and also reduce the diversity of bacterial communities in nearby waters and sediments.

## Figures and Tables

**Table 1:** Population size of various bacteria groups in sediment and water samples.

Sampling site	Total no. of heterotrophic bacteria (CFU/g or mL)	No. of ammonium-oxidizing bacteria (CFU/g or mL)	No. of nitrifying bacteria (CFU/g or mL)	No. of sulfate-reducing bacteria (cells/g or mL)	No. of nitrate-reducing bacteria (cells/g or mL)
Sediment of polluted sea area	6.70 ± 0.05 × 10^4^	1.90 ± 0.01 × 10^3^	9.80 ± 0.03 × 10^3^	4.60 ± 0.04 × 10^2^	1.20 ± 0.10 × 10^3^
Sediment of unpolluted sea area	4.30 ± 0.10 × 10^3^	1.50 ± 0.12 × 10^3^	4.60 ± 0.14 × 10^3^	2.30 ± 0.20 × 10^1^	2.10 ± 0.03 × 10^3^
Water of fish pond	1.25 ± 0.13 × 10^5^	7.50 ± 0.01 × 10^2^	4.40 ± 0.17 × 10^4^	<3	4.30 ± 0.20 × 10^3^
Water of effluent channel	1.29 ± 0.32 × 10^5^	2.10 ± 0.05 × 10^2^	4.90 ± 0.06 × 10^3^	<3	1.50 ± 0.09 × 10^4^
Water of polluted sea area	1.23 ± 0.15 × 10^4^	6.20 ± 0.08 × 10^2^	4.60 ± 0.09 × 10^3^	7.50 ± 0.21 × 10^1^	1.10 ± 0.05 × 10^3^
Water of unpolluted sea area	1.60 ± 0.08 × 10^3^	4.00 ± 0.20 × 10^1^	1.00 ± 0.05 × 10^2^	<3	9.00 ± 0.15 × 10^2^

**Table 2:** The Shannon index of the bacteria in the water and sediment samples shown by DGGE bands.

Sample no.	1	2	3	4	5	6
Shannon index	1.25	1.27	1.37	1.46	1.15	1.19

**Table 3:** Closest BLAST match for 16S rRNA genes of bacteria in fish-culture-associated environments.

Sampling sites	No. of DG E bands	Sizes of the DNA (bp)	Closest relative	Accession no. of BLAST closest match	% identity	Classification of strains
Sediment of polluted sea area	H1	408	*Gelidibacter* sp.	EF108219	99%	Flavobacteriaceae
	H2	415	*Psychroserpens mesophilus*	DQ001321	98%	Flavobacteriaceae
	H3	410	*Lacinutrix copepodicola*	AB261015	98%	Flavobacteriaceae
	H4	412	*Croceimarina litoralis*	EF108214	96%	Flavobacteriaceae
	H5	407	*Actibacter sediminis*	EF670651	100%	Flavobacteriaceae
	H6	415	*Maribacter polysiphoniae*	AM497875	98%	Flavobacteriaceae

Sediment of unpolluted sea area	I1	408	*Flavobacteriaceae bacterium*	EF527870	97%	Flavobacteriaceae
	I2	409	*Winogradskyella thalassocola*	AY771720	98%	Flavobacteriaceae
	I3	399	*Desulfuromonas *sp.	AY177801	97%	*δ*-proteobacterium
	I4	412	Uncultured deltaproteo- bacterium	DQ351798	99%	*δ*-proteobacterium

Water of fish ponds	J1	391	Thiotrophic endosymbiont of *Idas *sp.	AM402957	93%	Bacteria
	J2	410	*Zobellia laminariae*	AB121975	98%	Flavobacteriaceae

Water of effluent channel	K1	402	*Formosa *sp.	AY612758	97%	Flavobacteriaceae
	K2	405	*Winogradskyella thalassocola*	AY771720	97%	Flavobacteriaceae
	K3	397	*Polaribacter* sp.	AF493675	98%	Flavobacteriaceae
	K4	400	*Polaribacter dokdonensis*	DQ481463	98%	Flavobacteriaceae
	K5	411	*Marinobacter *sp.	DQ530471	98%	*γ*-proteobacteria
	K7	407	Uncultured* F. bacterium *	AM279213	98%	Flavobacteriaceae
	K8	409	*Pseudoalteromonas* sp.	EF673280	95%	*γ*-proteobacteria
